# Microglial *Pten* safeguards postnatal integrity of the cortex and sociability

**DOI:** 10.3389/fimmu.2022.1059364

**Published:** 2022-12-14

**Authors:** Xing Zhou, Jiacheng Wei, Liang Li, Zhenfeng Shu, Ling You, Yang Liu, Ruozhu Zhao, Jiacheng Yao, Jianbin Wang, Minmin Luo, Yousheng Shu, Kexin Yuan, Hai Qi

**Affiliations:** ^1^ Tsinghua-Peking Center for Life Sciences, Beijing, China; ^2^ Laboratory of Dynamic Immunobiology, Institute for Immunology, Tsinghua University, Beijing, China; ^3^ Department of Basic Medical Sciences, School of Medicine, Tsinghua University, Beijing, China; ^4^ Department of Neurology, Huashan Hospital, State Key Laboratory of Medical Neurobiology, Institute for Translational Brain Research, MOE Frontiers Center for Brain Science, Fudan University, Shanghai, China; ^5^ Department of Bioengineering, School of Medicine, Tsinghua University, Beijing, China; ^6^ IDG/McGovern Institute for Brain Research, Tsinghua University, Beijing, China; ^7^ School of Life Sciences, Tsinghua University, Beijing, China; ^8^ National Institute of Biological Science, Beijing, China; ^9^ Beijing Key Laboratory for Immunological Research on Chronic Diseases, Tsinghua University, Beijing, China; ^10^ Beijing Frontier Research Center for Biological Structure, Tsinghua University, Beijing, China

**Keywords:** microglia, PTEN, phagocytosis, VGLUT1, autism spectrum disorders, cerebral cortex, auditory cortex, layer 5

## Abstract

Microglial abnormalities may contribute to neurodevelopmental disorders. PTEN is implicated as a susceptibility gene for autism spectrum disorders and its germline ablation in mice causes behavioral abnormalities. Here we find postnatal PTEN deletion in microglia causes deficits in sociability and novel object recognition test. Mutant mice harbor markedly more activated microglia that manifest enhanced phagocytosis. Interestingly, two-week postponement of microglia PTEN ablation leads to no social interaction defects, even though mutant microglia remain abnormal in adult animals. Disturbed neurodevelopment caused by early PTEN deletion in microglia is characterized by insufficient VGLUT1 protein in synaptosomes, likely a consequence of enhanced removal by microglia. In correlation, *in vitro* acute slice recordings demonstrate weakened synaptic inputs to layer 5 pyramidal neurons in the developing cortex. Therefore, microglial PTEN safeguards integrity of neural substrates underlying sociability in a developmentally determined manner.

## Introduction

Microglia are tissue-resident macrophages in the central nervous system. Their progenitors develop in the yolk sac, migrate into the embryonic brain and by local proliferation populate the entire brain throughout life (1–4). Microglia participate in synaptic pruning, remodeling and maturation, particularly during postnatal neurodevelopment (5–7). Ablation of CX_3_CR1, a chemokine receptor that is expressed by microglia and mediates microglia-neuron interactions (8), leads to impaired synapse maturation, decreased functional brain connectivity, and autism-like abnormal behaviors (9, 10). Mice lacking the Triggering Receptor Expressed On Myeloid Cells 2 (TREM2), which in the brain is mainly expressed by microglia, exhibit a loss of microglia and abnormally increased spine densities selectively in the hippocampal CA1 region, and these mice suffer from behavioral defects (11). On the other hand, increasing evidence also indicates that, in adult brain, microglia actively probe neuronal states and play important roles in damage clearance, immune protection, trophic support and neuroplasticity (12–16). Phenotypic and functional abnormalities of microglia are intimately associated with neurodegenerative diseases. Prominently, in rodent models of neurodegenerative diseases including the Alzheimer’s disease (AD) and Amyotrophic lateral sclerosis (ALS), microglia display a stereotypic disease-associated microglia (DAM) phenotype (17), which appears to be an inflammatory state driven by a TREM2-dependent pathway (18). While the disease-associated microglia phenotype may or may not be a cause of pathogenesis, it is clear that changes to microglia, particularly those genetically determined, could lead to behavioral phenotypes either *via* a neurodevelopmental route in infants or children or *via* a functional route in adults.

The autism spectrum disorder (ASD) is a heterogenous group of neurodevelopmental disorders that are characterized by childhood-onset impairment of communication and social skills combined with the presence of repetitive behaviors. Genetic predisposition play a significant role, with ~1000 genes by some estimate potentially contributing to ASD susceptibility (19). Certain monogenic mutations greatly increase the risk of ASD, such as those affecting *FMR1* in fragile X syndrome (20, 21), *MECP2* in Rett syndrome (22), and *TSC1* and *TSC2* in Tuberous Sclerosis Complex (23). A commonly dysregulated pathway in these syndromic ASDs is the PI3 kinase and mTOR pathway (24). Dysregulation of PI3K and mTOR activities in neurons affects migration, axon formation and targeting, dendritic growth and spine development, potentially contributing to ASD pathogenesis directly (24). Phosphatase and Tensin Homolog (PTEN) is a tumor suppressor and a main lipid phosphatase that negatively regulates PI3K/Akt/mTOR pathways, which are vital to normal functions of essentially all cell types (25). Mutations of *PTEN* are found in ASD patients, particularly those with macrocephaly (26–30). In mice, Nse-cre-driven *Pten* deletion in a subset of neurons in the cortex and hippocampus leads to macrocephaly and deficits in social interactions reminiscent of ASD (31, 32), indicating a neuronal root of ASD pathogenesis due to loss of PTEN function. Given the fact that PTEN is universally expressed, other cell types may also contribute. Indeed, studies of *PTEN* mutations that cause cytoplasmic- or nuclear-predominant localization have revealed abnormalities of not only neurons but also glial cells, including microglia, in association with ASD-like behavioral phenotypes. In particular, dysregulated subcellular PTEN localization cause aberrant microglia activation with increased phagocytic activities (33, 34). Consistent with the idea that abnormal microglia functions may be involved in ASD pathogenesis due to *PTEN* mutations (33, 35–37), microglial overexpression of eIF4E leads to elevated protein synthesis and functional disturbance of microglia, altered synapse density, and ASD-like behaviors in mice (37). Our current study is designed to specifically probe how PTEN in microglia may regulate microglia-neuron crosstalk and whether microglial PTEN perturbation could affect neurodevelopment and animal behaviors. We report postnatal PTEN deletion in microglia immediately after birth leads to accumulation of activated microglia, accompanied by reduced synapse density and markedly changed firing patterns of cortical layer 5 (L5) pyramidal neurons, leading to impaired sociability later in life. Postponement of genetic deletion by 14 days completely avoided neuronal and behavioral impairment, demonstrating a developmental period during which intact microglia are essential for normal neural substrates underlying sociability.

## Results

### Postnatal *Pten* ablation in microglia leads to deficits in sociability and novelty exploration

**Figure 1:**
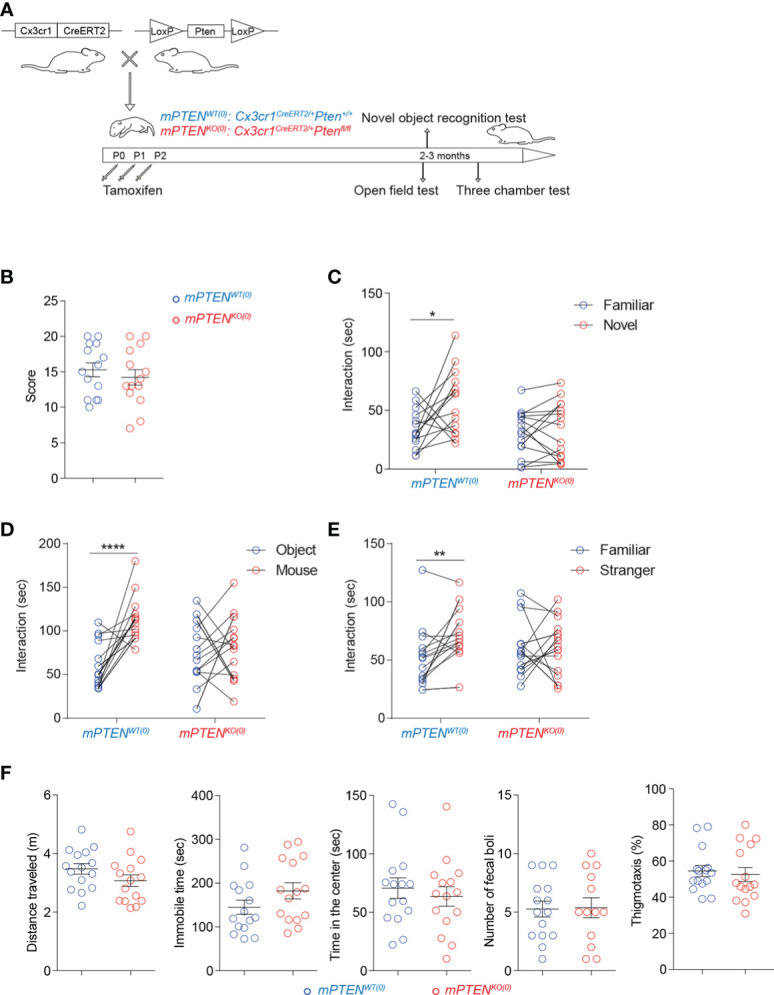
Postnatal *Pten* ablation in microglia leads to deficits in sociability and novelty exploration **(A)** Experimental scheme. **(B)** Numbers of marbles buried in 30 min during the marble-burying test (mPTEN^WT(0)^, n=14; mPTEN^KO(0)^, n=14). **(C)** Time spent exploring a novel versus a familiar object by mPTEN^WT(0)^ (n=14) or mPTEN^KO(0)^ (n=15) mice in the novel object recognition test. **(D)** Time spent interacting with an inanimate object or a mouse by mPTEN^WT(0)^ (n=17) or mPTEN^KO(0)^ (n=17) mice in the three-chamber interaction test. **(E)** Time spent interacting with a familiar or a stranger mouse by mPTEN^WT(0)^ (n=17) or mPTEN^KO(0)^ (n=17) mice in the three-chamber test. **(F)** Locomotor activities and thigmotaxis in the open-field test. Total distance traveled (m), total time of being immobile (sec), total time of being in the center (sec), number of fecal boli left and thigmotaxis by individual mPTEN^WT(0)^ (n=15) and mPTEN^KO(0)^ (n=15) mice. *P < 0.05, **P < 0.01, ****P < 0.0001, by unpaired (open field and marble-burying) or paired (novel objection recognition and three-chamber interaction) *t* tests.

To investigate a potential role for PTEN in microglia in shaping animal behaviors, we created mice with inducible *Pten* deletion in a microglia-specific manner by breeding *Cx3cr1* ^CreERT2/+^ mice with *Pten* ^fl/fl^ mice. To specifically examine PTEN-controlled microglial functions during postnatal neurodevelopment, as detailed in Methods and illustrated in **Figure 1A**, we injected tamoxifen into the stomach of newborn *Cx3cr1* ^CreERT2/+^ *Pten* ^fl/fl^ and littermate control *Cx3cr1* ^CreERT2/+^ *Pten* ^+/+^ mice daily from postnatal day 0 (P0) to day 2 (P2). In this system, microglia and other cells in the brain are intact throughout embryonic development. To verify the efficiency of *Pten* deletion by this procedure, microglia were isolated at different time points to test PTEN protein expression. As compared to cells from littermate control mice, microglia from *Cx3cr1* ^CreERT2/+^ *Pten* ^fl/fl^ mice largely lost PTEN protein as early as one week after the tamoxifen injection and remained so for as long as we tested (**Figures S1A, B**). Taking advantage of the EYFP expression from the knock-in *Cx3cr1* ^CreERT2^ allele, we used anti-GFP antibody to stain for EYFP on tissue sections and verified that the targeted cells were essentially all Iba-1^+^ microglia but not astrocytes (S100b^+^), oligodendrocytes (Olig2^+^), or neurons (NeuN^+^) (**Figures S1C, D**). For convenience, we subsequently call tamoxifen-treated *Cx3cr1* ^CreERT2/+^ *Pten* ^fl/fl^ and *Cx3cr1* ^CreERT2/+^ *Pten* ^+/+^ mice mPTEN^KO(0)^ and mPTEN^WT(0)^, respectively.

To examine behavioral consequences of postnatal PTEN deletion in microglia, we first compared mPTEN^KO(0)^ and mPTEN^WT(0)^ mice (>8 weeks old) in a marble-burying test, which could reveal signs of ASD-like repetitive behaviors and potentially increased anxieties (38). As shown in **Figure 1B**, the two groups of mice were comparable. On the other hand, when tested in a novel object recognition test (NORT), mPTEN^KO(0)^ mice failed to exhibit a preference for exploring the novel object, unlike their mPTEN^WT(0)^ counterparts (**Figure 1C**). In social interaction tests, whereas mPTEN^WT(0)^ mice showed a clear preference for interacting with a companion mouse than with an inanimate object (**Figure 1D**) and for interacting with a stranger than with a familiar mouse (**Figure 1E**), the mPTEN^KO(0)^ mice did not show such preferences (**Figures 1D, E**). The failure of mPTEN^KO(0)^ mice to pass these tests did not result from grossly impaired locomotor abilities or increased anxieties, as they performed as well as the wildtype counterparts in the open-field test (**Figure 1F**). These data demonstrate impaired sociability and exploration of novel objects resulting from postnatal PTEN ablation in microglia.

### Postnatal *Pten* ablation leads to accumulation of activated microglia

**Figure 2:**
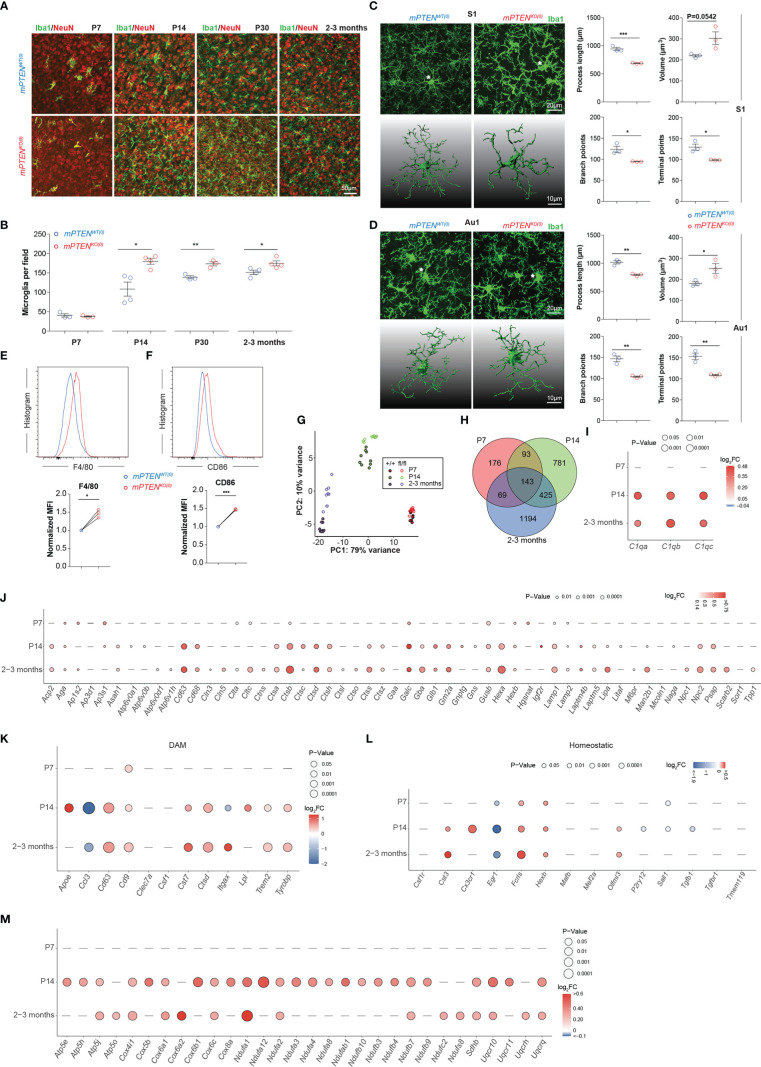
Microglia phenotypes after postnatal *Pten* ablation. **(A)** Representative images of Iba1-stained microglia (green) in the context of NeuN staining (red) in the somatosensory cortex (coronal sections) of mPTEN^WT(0)^ and mPTEN^KO(0)^ mice at indicated time points. **(B)** Numbers of Iba1^+^ microglia per field (0.4 mm^2^), as quantified from **(A)**. Each dot represents a mouse (microglia quantitated in n=5-10 fields per mouse), and lines denote mean±s.e.m. (P7, P14, P30, and 2-3 months, N=3, 4, 3, 3 mice). **(C)** Left, maximum intensity projection images (top) and 3-D reconstruction of the asterisked microglia (bottom) from the somatosensory cortex (S1) of 2- to 3-month-old mice, mPTEN^WT(0)^ or mPTEN^KO(0)^ as indicated; Right, automated morphometric analyses of microglial process length, volume, number of branch points, and number of dendrite terminal points. Each dot represents a mouse (5-10 cells per mouse), and lines denote mean±s.e.m. of individual mice. **(D)** Microglia from the auditory (Au1) cortex, analyzed and presented exactly as in **(C)**. **(E, F)** Representative histograms (top) and normalized MFI (bottom) of F4/80 **(E)** and CD86 **(F)** staining of mPTEN^WT(0)^ and mPTEN^KO(0)^ microglia (2- to 3-month-old mice). Each pair of line-connected dots represents one of the three independent experiments conducted. In each experiment, microglia of each genotype were pooled from 2 mice. *P < 0.05, **P < 0.01, ***P < 0.001 by unpaired *t* test **(B-F)**. **(G)** Transcriptomic PCA analyses of sort-purified cortical microglia from P7, P14 and 2-3 months. **(H)** Venn diagram of DEGs between wildtype and PTEN-ablated microglia at P7, P14 to 2-3 months. **(I)** A bubble plot showing differential expression of indicated complement genes (X axis) at indicated time points (Y axis). Bubble color, Log_2_FC=Log_2_(mPTEN^KO(0)^/mPTEN^WT(0)^); bubble size, levels of statistical significance defined by *P* values; a dash indicates no significant difference observed. **(J)** A bubble plot showing differential expression of lysosome-related genes at indicated time points, formatted as in **(I)**. **(K, L)** A bubble plot showing differential expression of indicated DAM **(K)** and homeostatic **(L)** genes, formatted as in **(I)**. **(M)** A bubble plot showing differential expression of indicated mitochondria-related genes, formatted as in **(I)**.

To characterize changes of postnatal microglial development and function caused by *Pten* ablation, we first examined their progressive expansion. We chose the cerebral cortex for two reasons. First, microglia from different brain regions are quite heterogeneous (39), and cerebral cortex represents an abundant source of microglia. Second, cortical functions are important for social behaviors (40, 41). As shown in **Figures 2A, B**, the density of Iba-1^+^ microglia gradually increased to reach a stable plateau by 2-3 months after birth (hereafter “young adult” is used to specifically refer to 2- to 3-month-old mice), with the most rapid expansion taking place during the second week of postnatal development. When *Pten* was ablated from microglia, these cells accumulated almost twice as fast to reach approximately the plateau density by P14; in young adult mice or older animals, the density of mPTEN^KO(0)^ microglia in cerebral cortex was only slightly albeit statistically significant higher than that of mPTEN^WT(0)^ cells (**Figures 2A, B**). We did not observe differences in density or distribution of neurons, astrocytes, or oligodendrocytes in the two types of mice (**Figure S1E**). Individual mutant microglia exhibited markedly different morphology in that their cell bodies were enlarged, with thickened cellular processes extending shorter and fewer branches, indicative of an activated state (42), as seen in both somatosensory (**Figure 2C**) and auditory cortices (**Figure 2D**). Consistent with these morphological patterns, mPTEN^KO(0)^ microglia from young adult mice expressed higher levels of F4/80 and CD86 (**Figures 2E, F**), which are upregulated in activated microglia (43).

Next, we conducted transcriptomic profiling of mPTEN^WT(0)^ and mPTEN^KO(0)^ microglia isolated from postnatal day 7 (P7) mice, postnatal day 14 (P14) mice and 2- to 3-month-old young adult mice. By principal component analyses, we found that mPTEN^WT(0)^ and mPTEN^KO(0)^ microglia from the same time of postnatal development were grouped together, whereas microglia of the same genotype, either mPTEN^WT(0)^ or mPTEN^KO(0)^, displayed divergent transcriptomes across different time points of postnatal development (**Figure 2G**). Therefore, while microglia-intrinsic *Pten* deletion led to changes in cell abundance and morphology, it did not completely alter the overall trajectory of postnatal microglial development, which involves progressive changes of the transcriptome at a larger scale (44).

At a finer scale, on the other hand, the number of genes differentially expressed by wildtype and mPTEN^KO(0)^ microglia increased over time (**Figure 2H**, **Table S1**). Prominently, genes encoding C1q components (*C1qa*, *C1qb*, and *C1qc*) were significantly upregulated in mutant microglia at P14 and later (**Figure 2I**). We also observed increased C1q protein by immunostaining on tissue sections (**Figures S2A, B**). Many lysosome-related genes (as defined by the KEGG pathway), including *Cd68* and those encoding cathepsins, were expressed at significantly higher levels in PTEN-deficient microglia at P14 and later (**Figure 2J**). Interestingly, many genes upregulated in the DAM signature, which reflects an inflammatory microglia state observed in neurodegenerative diseases including AD and ALS (17, 18), were also expressed at higher levels in mPTEN^KO(0)^ than in mPTEN^WT(0)^ microglia at P14 and later (**Figure 2K**). On the other hand, general downregulation of microglia homeostatic genes (e.g. *P2ry12*, *Cx3cr1*, *Cst3*, *Tmem119*, *Olfml3*, *Hexb*, *Fcrls*, *Csf1r*, *Tgfbr1*, *Mef2a*, *Mafb*) seen in the DAM signature (44–46) did not occur, as expression of most of these genes were either unchanged or increased in mPTEN^KO(0)^ microglia (**Figure 2L**). Gene set enrichment analyses further revealed that, at all three time points examined, genes upregulated in mPTEN^KO(0)^ microglia enriched those in KEGG pathways of oxidative phosphorylation, AD, and the Parkinson’s disease (PD; **Figures S3A-C**). A shared feature of these three KEGG pathways is that they all contain many genes encoding mitochondrial components (**Table S2**), reflecting the fact that mitochondria dysfunctions are implicated in the pathogenesis of AD and PD (47). As shown in **Figure 2M**, many mitochondria-related genes categorized in the 3 pathways are expressed at higher levels in mPTEN^KO(0)^ than in mPTEN^WT(0)^ microglia from P14 or young adult mice.

Combined together, these profiling data suggest that PTEN ablation leads to an activated microglia state that features enhanced oxidative phosphorylation, increased phagocytosis, and possibly altered C1q-dependent synapse pruning (48).

### Microglia without PTEN are more phagocytic

**Figure 3:**
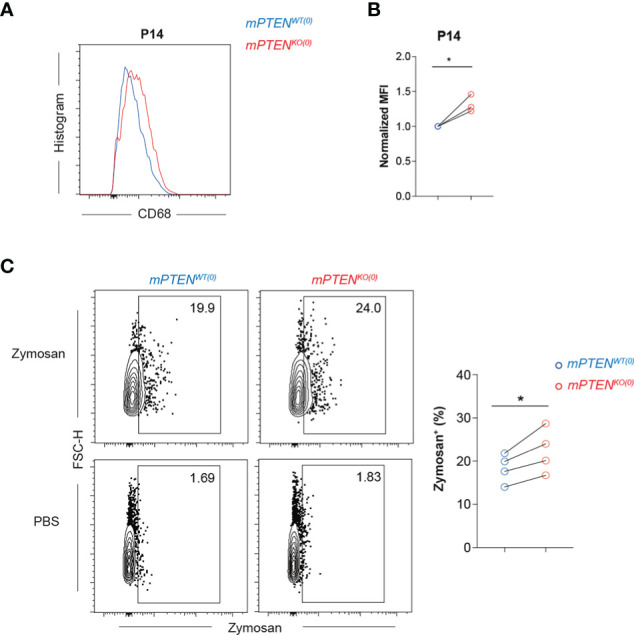
Microglia without PTEN are more phagocytic **(A, B)** Histograms **(A)** and normalized MFI **(B)** of CD68 staining on mPTEN^WT(0)^ (blue) and mPTEN^KO(0)^ (red) microglia at P14. Each pair of line-connected dots represents one of the three independent experiments conducted. In each experiment, cortical microglia of each genotype were pooled from 2 male mice. **(C)** Contour plots showing freshly isolated microglia at P7 that were incubated *in vitro* with 0.2 mg/ml zymosan or PBS for 1.5 h. The scatter plot shows summary data of the percentage of zymosan-containing microglia of the indicated genotype. Each dot represents one experiment, and in each experiment microglia of cerebral cortex were pooled from 4-5 male mice. Lines denote mean ± s.e.m. *P < 0.05, by paired t test.

Phagocytosis is important for microglial homeostatic and defense functions (49, 50). PTEN could directly regulate phagosome membrane trafficking, because its enzymatic activities reduce membrane PIP3 concentration (51, 52). KEGG pathway enrichment analysis of upregulated genes in mPTEN^KO(0)^ microglia reveals lysosome pathway is most significantly enriched at all three time points examined (**Figure S3D**). Consistent with the mRNA-seq results (**Figure 2J**), mPTEN^KO(0)^ microglia expressed higher levels of intracellular CD68 protein at P14 (**Figures 3A, B**). By flow cytometry, freshly isolated mPTEN^KO(0)^ microglia were able to phagocytose more zymosan particles *in vitro* (**Figure 3C**), indicating that the intrinsic phagocytic capability is enhanced in PTEN-deficient microglia.

### A critical window for microglia PTEN to shape neural development underlying sociability

To directly test whether PTEN deletion from microglia earlier in postnatal life is causative for defective sociability observed later in life, we injected tamoxifen into separate groups of *Cx3cr1* ^CreERT2/+^ *Pten* ^fl/fl^ and littermate control *Cx3cr1* ^CreERT2/+^ *Pten* ^+/+^ mice on postnatal day 14 to create mPTEN^KO(14)^ and mPTEN^WT(14)^ mice. PTEN deletion was verified by Western blotting with sorted microglia, as done in **Figure S1B**.

**Figure 4:**
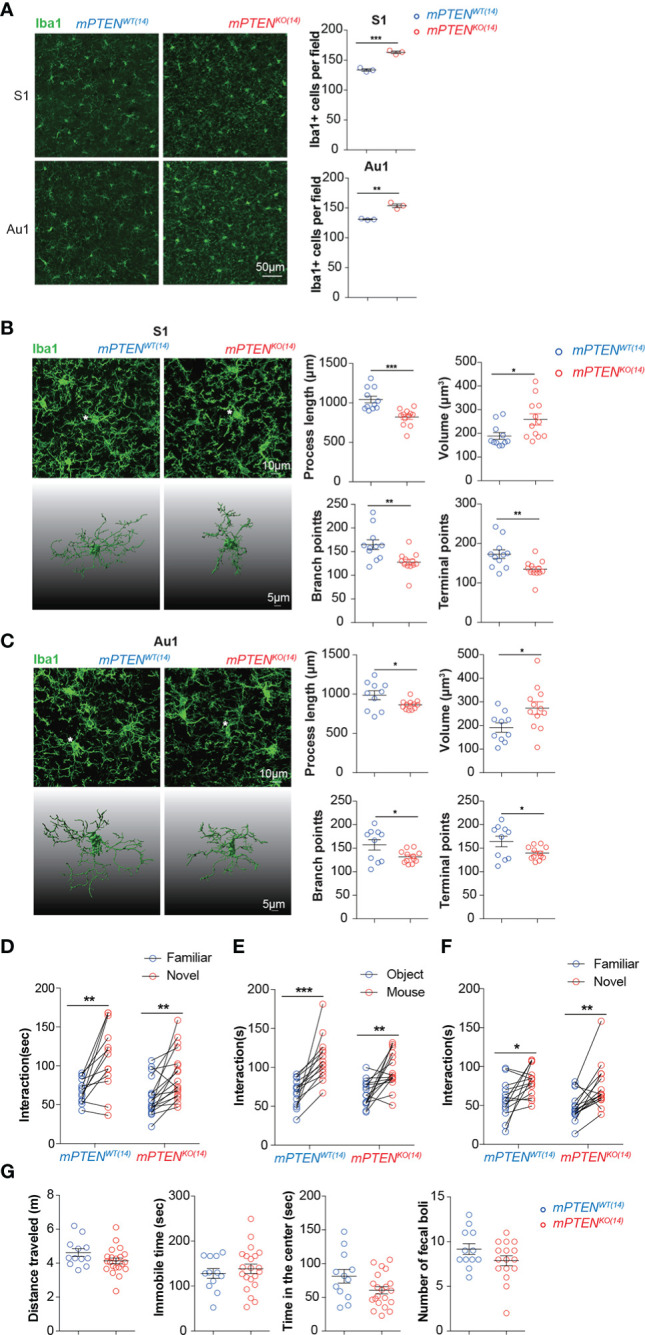
A critical window for microglia PTEN functions. **(A)** Representative images of Iba1-stained microglia in the S1 and Au1 cortex of mPTEN^WT(14)^ and mPTEN^KO(14)^ mice of 2-3 months of age. Right: numbers of Iba1^+^ microglia per field (0.4 mm^2^). Each dot represents a mouse (3 fields per mouse), and lines denote mean±s.e.m. (3 mice). **(B)** Left, maximum intensity projection images (top) and 3-D reconstruction of the asterisked microglia (bottom) from the somatosensory cortex (S1) of 2- to 3-month-old mice, mPTEN^WT(14)^ or mPTEN^KO(14)^ as indicated; Right, automated morphometric analyses of microglial process length, volume, number of branch points, and number of dendrite terminal points. Each dot represents one 3-D reconstructed microglia, and lines denote mean ± s.e.m. Data were collected from 3 mice, each contributing 3-4 randomly chosen microglia in the S1 cortex. **(C)** Microglia from the auditory (Au1) cortex, analyzed and presented exactly as in **(B)**. *P < 0.05, **P < 0.01, ***P < 0.001, by unpaired *t* tests. **(D)** Time spent exploring a novel versus a familiar object by mPTEN^WT(14)^ (n=13) or mPTEN^KO(14)^ (n=18) mice in the novel object recognition test. **(E, F)** Time spent interacting with an inanimate object or a mouse **(E)**, a familiar or a stranger mouse **(F)** by mPTEN^WT(14)^ (n = 14) or mPTEN^KO(14)^ mice (n=16) in the three-chamber test. **(G)** Locomotor activities in the open-field test. Total distance traveled (m), total time of being immobile (sec), total time of being in the center (sec), and number of fecal boli left by individual mPTEN^WT(14)^ (n = 12) and mPTEN^KO(14)^ (n = 12) mice. *P < 0.05, **P < 0.01, ***P < 0.001 by unpaired (open field) or paired (novel objection recognition and three-chamber interaction) *t* tests.

Interestingly, microglia remained of overactive features in young adult mPTEN^KO(14)^ mice. Mutant microglia were more abundant than their wildtype counterpart (**Figure 4A**), and their cellular processes were thickened and shorter with fewer branches (**Figures 4B, C**). The transcriptome of those mPTEN^KO(14)^ microglia was globally similar to that of microglia from mPTEN^KO(0)^ mice (**Figure S4A**), which is consistent with the PCA result for mPTEN^(0)^ mice that microglia from the same age tend to cluster together (**Figure 2G**). Genes encoding C1q components (*C1qa*, *C1qb*, and *C1qc*) were also upregulated in mPTEN^KO(14)^ microglia (**Figure S4B**).

Despite those similar abnormalities between mPTEN^KO(14)^ and mPTEN^KO(0)^ microglia in adult animals, mPTEN^KO(14)^ mice performed normally in novel object recognition and social interaction tests (**Figures 4D, G**). Further transcriptome analysis showed that the number of differentially expressed genes between mPTEN^KO(14)^ and mPTEN^WT(14)^ microglia in young adult mice was greatly reduced, compared to that observed between mPTEN^KO(0)^ and mPTEN^WT(0)^ microglia (1411 vs 1831, **Table S3**). Fewer lysosome genes were upregulated in mPTEN^KO(14)^ microglia as compared to those upregulated in mPTEN^KO(0)^ microglia (**Figure S4C**, **Figure 2J**). GSEA analysis indicates that genes differentially expressed between mPTEN^KO(14)^ and mPTEN^WT(14)^ microglia were no longer enriched with KEGG pathways implicated in AD and PD, although those implicated in oxidative phosphorylation were still enriched (**Figure S4D**). Consistently, the number of enriched KEGG pathways was dramatically decreased compared to that enriched in mPTEN^(0)^ mice, although lysosome pathway was still enriched (**Figure S4E**). Most DAM signature genes and mitochondria-related genes that were upregulated in mPTEN^KO(0)^ microglia were not differentially expressed in mPTEN^KO(14)^ microglia (**Figures S4F, G**).

Taken together, these data indicate that the first 2 weeks are the most critical period during which PTEN in microglia must be intact in order to safeguard brain circuit development underlying normal sociability.

### Impaired neurodevelopment in mPTEN^KO(0)^ mice

**Figure 5:**
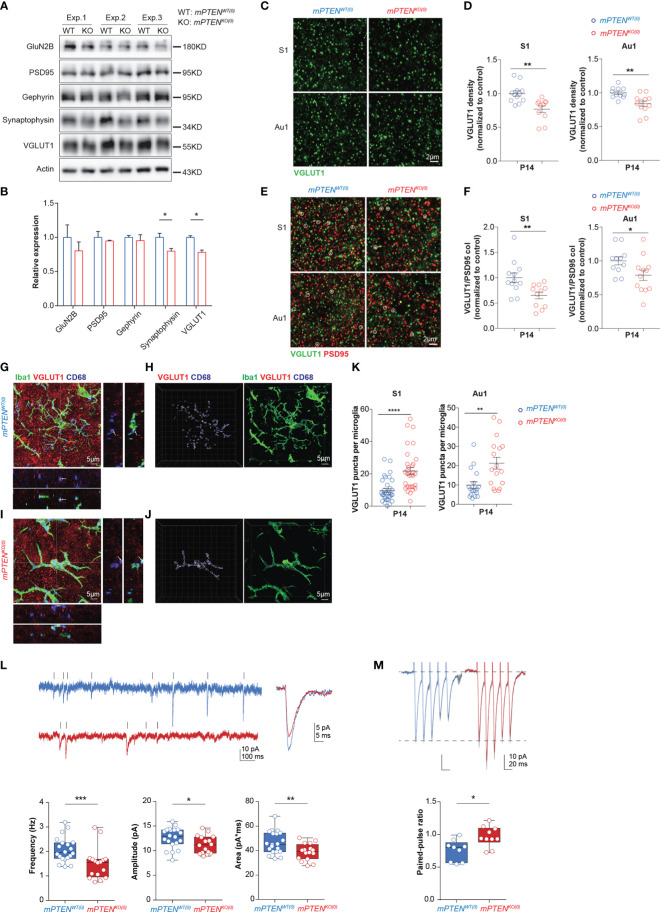
Defects of mPTEN^KO(0)^ mice at P14. **(A, B)** Western blotting of presynaptic proteins (VGLUT1, synaptophysin), postsynaptic proteins (PSD95, GluN2B), and inhibitory synaptic protein Gephyrin in 2-3-month cortex **(A)** and actin-normalized expression levels **(B)**. Bar graphs show mean±s.e.m. of the three (Exp. #1, #2, #3) experiments. *P < 0.05 by unpaired *t* test. **(C, D)** Representative images of VGLUT1 staining of the S1 and Au1 cortex **(C)**, and scatter plots showing densities of VGLUT1 puncta in S1 and Au1 cortex **(D)**. **(E, F)** Representative images of VGLUT1 and PSD95 staining of the S1 and Au1 cortex, with VGLUT1/PSD95 co-localized puncta highlighted with circles **(E)**, and scatter plots showing densities of VGLUT1/PSD95 puncta in the S1 and Au1 cortex **(F)**. Data were collected from 3 mice per genotype (for each mouse, 5 fields in S1 and 5 fields in Au1 cortex). Each symbol is one field, and lines denote mean ± s.e.m. *P<0.05, **P<0.01, by unpaired *t* tests. **(G-J)** Maximum intensity projection image of an mPTEN^WT(0)^ **(G)** or mPTEN^KO(0)^ **(I)** Iba-1^+^ (green) microglia that contains VGLUT1^+^ (red) puncta within CD68^+^ vesicles (blue). XZ and YZ views show localization of VGLUT1 puncta within CD68 vesicles at crossing points highlighted in projection images. 3-D views of the images in **(G, I)** are shown in **(H, J)**, respectively, with VGLUT1, CD68 and Iba-1 staining rendered as iso-surfaces in Imaris. **(K)** Numbers of VGLUT1 puncta per microglia in S1 (left) and Au1 (right) cortex. Each symbol in scatter plots represents one 3-D reconstructed microglia, and lines denote mean ± s.e.m. Data were collected from 3 P14 mice of each genotype, each contributing 5-6 randomly chosen microglia in the S1 and Au1 cortex, respectively. **P < 0.01, ****P < 0.0001, by unpaired *t* tests. **(L)** Example recordings of mEPSCs of L5 pyramidal neurons from P14-16 mPTEN^WT(0)^ and mPTEN^KO(0)^ brain slices, and the average mEPSC amplitude, frequency and area (mPTEN^WT(0)^: n=20, 3 mice; mPTEN^KO(0)^: n=16, 3 mice). Mann-Whitney tests for amplitude and area, unpaired *t* test for frequency. **(M)** Examples of evoked EPSCs and quantification of the paired-pulse ratio of the evoked EPSCs (mPTEN^WT(0)^: n=9, 3 mice; mPTEN^KO(0)^: n=10, 3 mice); unpaired *t* test. *P < 0.05, **P < 0.01, ***P < 0.001.

Because synapses and circuit connections are often altered in ASD (53–55), we further examined synaptic changes as a result of microglial PTEN deletion. We prepared synaptosomes from 2-3-month cerebral cortex and probed different presynaptic and postsynaptic components by Western blotting. A significant reduction in presynaptic VGLUT1 and synaptophysin was observed in mPTEN^KO(0)^ as compared to control synaptosomes, whereas postsynaptic protein PSD95, the NMDA receptor subunit GluN2B, and Gephyrin were not appreciably altered (**Figures 5A, B**). By immunostaining of tissue sections, we found that the density of VGLUT1 puncta was significantly reduced in deep layers of somatosensory and auditory cortices in P14 mPTEN^KO(0)^ mice (**Figures 5C, D**). Excitatory synapses, as identified by colocalized VGLUT1 and PSD95 staining, showed a significantly reduced density in these mutant mice (**Figures 5E, F**). To investigate whether the reduction of presynaptic VGLUT1 correlates with more phagocytic microglia at P14, we examined VGLUT1 staining together with Iba-1 and CD68. As shown in **Figures 5G-K**, Iba-1^+^ microglia in P14 mPTEN^KO(0)^ L5 contained more VGLUT1 puncta within CD68^+^ vesicles. These data strongly suggest that mutant microglia has already been more actively removing VGLUT1-containing pre-synapses in the early developmental stage.

Given perturbed microglia-synapse interactions observed (mostly L5), we explored early-life functional changes of neurons by electrophysiology. Disrupted intrahemispheric and intracortical functional connectivity are implicated in abnormal social behaviors (56), and L5 pyramidal neurons account for the largest fraction of intracortical output (57). Degraded auditory information processing and transfer would impair speech recognition and communication in human (58, 59). We therefore conducted *in vitro* recordings from L5 pyramidal neurons in the auditory cortex. Consistent with the decrease of VGLUT1 contents and reduction of excitatory synapses in mPTEN^KO(0)^ cortex, whole-cell patch clamp recordings from L5 pyramidal neurons in acute slices revealed markedly reduced frequency, amplitude and area of miniature excitatory postsynaptic currents (mEPSC) in L5 pyramidal neurons in P14 auditory cortex (**Figure 5L**). Furthermore, paired-pulse ratios were significantly increased in mPTEN^KO(0)^ L5 pyramidal cells (**Figure 5M**), suggesting a decrease in release probability of glutamate vesicles from presynaptic terminals. Because levels of postsynaptic AMPA receptor GluA1 and GluA2 subunits showed no difference in synaptosomes of P14 mPTEN^KO(0)^ and mPTEN^WT(0)^ mice (data not shown), the decreased mEPSC amplitude potentially results from reduced quantal size (i.e. less glutamate in presynaptic vesicles) due to the reduced level of VGLUT1 expression (60).

These data support the possibility that defects introduced during the critical period of postnatal neurodevelopment underlie behavioral abnormalities later in life.

### Normal neuronal properties in young adult mPTEN^KO(14)^ mice

Consistent with normalized social behaviors of mPTEN^KO(14)^ mice, synaptosomes from the mPTEN^KO(14)^ cerebral cortex contained normal amounts of all presynaptic and postsynaptic proteins tested, including VGLUT1 (**Figures S5A, B**). mPTEN^KO(14)^ cortices in young adult mice had a similar level of VGLUT1 puncta (**Figures S5C, D**), and the density of VGLUT1^+^PSD95^+^ synapses was no longer reduced (**Figures S5E, F**). Therefore, when postponed beyond 2 weeks after birth, PTEN deletion in microglia does not cause neuronal and behavioral abnormalities as seen with earlier deletion.

## Discussion

Microglia play an important role in synaptic pruning for normal brain development (50). In the well-studied climbing fiber of the cerebellum and the dorsal lateral geniculate nucleus of the visual system, critical pruning takes place during the early postnatal period in rats and mice (61–63). During this critical period of neurodevelopment, microglia remove weaker presynaptic inputs in the dorsolateral geniculate nucleus in a complement-dependent manner (7). Our study demonstrates that microglia-specific *Pten* deletion, when induced immediately after birth but not after two weeks, causes profound defects in neuronal development and functions, leading to abnormal social behaviors later in life. The most likely explanation for this narrow window of effect is that certain circuits underlying social behaviors are abnormally sculpted by mutant microglia in the first 2 weeks or so after birth. Indeed, without PTEN, microglia become intrinsically more phagocytic, express more C1q components, and expand much more during the first 2 weeks postnatally. These effects in combination cause inappropriately exaggerated removal of synapses. Beyond 2 weeks after birth, even though PTEN deletion still causes increased phagocytosis and complement expression by microglia, circuits critical for social behaviors are likely already established and could not be fundamentally altered. Given the complexity of social behaviors, it is at present difficult to pinpoint particular circuitries that are being altered by mutant microglia. It is tempting to speculate that, as circuits are being established during the critical period of neurodevelopment, abnormally pruned connections have a propagating negative impact on subsequent circuit formation in a feedforward manner. Behaviors that depend on complex circuitries are probably vulnerable to abnormal pruning during the critical period. Given the well-documented implications of PTEN *de novo* mutations in human ASD (26–30), microglial abnormalities caused by an intrinsic lack of PTEN regulation of phagocytosis and cell expansion could be one contributing factor for the ASD pathogenesis.

Mice carrying germline loss-of-function alleles of *Cx3cr1* or *Trem2*, two genes with microglia-restricted expression in the central nervous system, exhibit defects in sociability (10, 11). Specifically, CX_3_CR1 deficiency causes decreased microglia numbers within the first month of life and a transient increase in dendritic spine density between the second to third postnatal week in the hippocampus (5), leading to immature synaptic multiplicity that persists into the adulthood (10). The triggering receptor expressed on myeloid cells 2 (TREM2) is a phagocytic receptor implicated in numerous neurodegenerative disorders (64). Hippocampal microglia from TREM2-deficient mice are numerically reduced and impaired in phagocytosis, while CA1 neurons exhibit an increased spine density in 3- but not 13-week-old mice (11). In both models, functional connectivity among different brain regions is reduced (10, 11), consistent with an association between ASD and long-range functional disconnections (65, 66). An interesting contrast provided in our study is that enhanced phagocytosis by a markedly enlarged population of PTEN-deficient microglia causes a specific decrease of VGLUT1-containing excitatory synapses. These changes are evident by 2 weeks with markedly altered electrophysiological properties of L5 pyramidal neurons, underlying defective social interactions later in life.

PTEN counteracts PI3K signaling, which is required for phagocytosis and would be triggered downstream of chemokine receptors including CX_3_CR1. It is therefore somewhat surprising that we only observed defects in social interactions and novel object recognition but not the wider range of additional behavioral abnormalities of TREM2- or CX_3_CR1-deficient mice (11, 67). CX_3_CR1 and TREM2 likely trigger additional signaling pathways, and it is an intriguing possibility that distinct receptors and signaling processes in microglia may differentially control sculpture of different circuits in various brain regions. On the other hand, in contrast to our time-resolved, cell-specific, inducible gene perturbation strategy, CX_3_CR1- and TREM2-deficient models previously studied invoke genetic lesion in the germline. It is less clear how CX_3_CR1 and TREM2 functions in embryo and how impairment of those embryonic functions might impact on microglia-mediated pruning postnatally. We speculate that, when examined in a time- and cell type-resolved manner, other genes associated with neurodevelopmental disorders may be found to contribute to disease phenotypes also *via* microglia in addition to neuron-intrinsic routes. By the same token, whereas our study can pinpoint the importance of microglial PTEN-dependent functions in the first two postnatal weeks, effects of PTEN loss-of-function in embryonic microglia remain unknown, and such effects might also be relevant to aspects of ASD pathogenesis in patients carrying PTEN mutations.

Beyond implications for ASD, it is interesting to note that when PTEN is deleted immediately after birth, microglia at P14 and the young adult stage show signs of the DAM signature, which is initially defined in the context of AD and ALS (17, 18). On the other hand, when PTEN deletion is postponed for 2 weeks, the DAM signature becomes much less evident in adult microglia. This is in contrast to genes in the lysosome or oxidative phosphorylation pathways and genes encoding complement components, which are expressed at increased levels by adult microglia no matter whether PTEN is deleted earlier or late. These observations suggest that PTEN deletion *per se* does not intrinsically lead to a DAM signature. Instead, the disease state of the brain caused by postnatal PTEN deletion in microglia likely makes a significant and reciprocal impact on microglia to induce a DAM-like state. In the same vein, even though PTEN protein is completely lost already by P7 when the *Pten* gene is deleted immediately after birth, those mitochondria-related genes implicated in oxidative phosphorylation, AD and PD (**Figure 2M**) are not significantly changed until P14, when neuronal abnormalities are evident (**Figure 5**). Again, it appears that neuronal abnormalities as an external force significantly affect microglia and contribute to their altered gene expression program. Deciphering complex interactions between neurons and microglia and their reciprocal regulation is likely a key to understanding of nervous system diseases.

## Materials and methods

### Mice

*Pten* ^fl/fl^ (Jax 006440), *Cx3cr1* ^CreER^ (Jax 021160), Thy1-YFP (Jax 003782) were originally purchased from the Jackson Laboratory. All mice were maintained on the C57BL/6 background, and were maintained under specific pathogen-free conditions. All mice analyzed were male mice. All animal experiments were conducted in accordance of government and Tsinghua guidelines for animal welfare and approved by Tsinghua Institution Animal Care and Use Committee.

### Postnatal *Pten* gene ablation in microglia

To induce *Pten* deletion, newborn littermates derived from a *Cx3cr1* ^CreER/CreER^ *Pten* ^fl/+^×*Cx3cr1* ^+/+^ *Pten* ^fl/+^ breeding scheme were given 50 μg tamoxifen in sunflower oil (Sigma) daily on P0, P1 and P2 (68). Those of the desired *Cx3cr1* ^CreER/+^ *Pten* ^fl/fl^ and *Cx3cr1* ^CreER/+^ *Pten* ^+/+^ genotypes were subsequently used as mPTEN^KO(0)^ and mPTEN^WT(0)^, respectively. For more delayed postnatal *Pten* deletion, littermates derived from the same breeding scheme were given two doses of 50 mg/kg body weight by gavage on P12 and P14 to produce mPTEN^WT(14)^ and mPTEN^KO(14)^ mice.

### Behavioral analyses

Young adult male mice of 2 to 3 months of age were used for all behavioral tests.

#### The marble-burying test

The marble-burying test was performed in novel cages (390×200×172 cm) containing 5 cm-thick fresh bedding materials. Twenty glass marbles (16 mm in diameter) were arranged evenly on the surface of the bedding in a 4×5 format. Each mouse was allowed to freely explore for 30 min before the number of buried marbles was counted, which were those with at least two thirds covered with beddings.

#### The open-field test

The open-field test was performed in an open arena (40×40×40 cm). Each mouse was allowed to freely explore for 10 min. Mouse movement was recorded by a video camera and analyzed by Any-maze software. Occasionally, some animals did not attempt to explore at all and were excluded from analysis. The basal locomotor activity was evaluated by the total distance traveled, and the anxiety level was evaluated by the total time immobile, total time spent in the center area (20×20 cm), and the number of fecal boli left during the test.

#### The novel object recognition test

The novel object recognition test was performed in an open arena (40×40×40 cm). Individual mice were habituated to the arena for 10 min. Twenty-four hours after the habituation, individual mice were allowed to explore the arena with two identical objects for 10 min (training phase); 30 min later, the mice were reintroduced into the arena for 10 min with one of the objects replaced with a novel one (test phase). Mice were considered to explore the object when reaching within 3 cm to the object. Exploration time was calculated by the Any-maze software.

#### The social interaction test

The social interaction test was performed in a three-chamber apparatus (60×45×25 cm). Mice were individually habituated to the chamber for 10 min. Twenty-four hours after the habituation, test mice were individually placed back into the middle chamber, while each of the side chambers contains a plastic cage, one empty and one housing an unfamiliar mouse of the same sex; interactions were analyzed for 10 min, and interaction time (within 3 cm to either cage) was calculated by the Anymaze software. Thirty minutes later, mice were reintroduced into the chamber for 10 min, with one cage housing the previously encountered mouse and the other housing a stranger mouse, and interaction analysis was repeated. Occasionally, some animals did not attempt to explore any chamber in the two tests above and were excluded from analysis.

### Isolation and flow cytometry analysis of microglia

Mice were anesthetized and transcardiacally perfused with PBS, the brains were quickly removed and the cortex were isolated in ice-cold HBSS (Gibico). The cortex was homogenized with the Kimble Dounce tissue grinder and then subjected to centrifugation through 30%/70% discontinuous Percoll (GE) gradient. The microglia-enriched fraction at the interface was washed with ice-cold PBS and then resuspended in the MACS buffer (PBS supplemented with 1% FBS and 5mM EDTA) for subsequent procedures. The cells were blocked for 10 min with Fc-blocker (Clone 2.4G2: 20 μg/ml; BioXcell), and then were incubated with the following antibodies for 30 min on ice: PerCPcy5.5 anti-CD45 (104), PE anti-CD86 (GL-1) from Biolegend, PE-Cy7 anti-CD11b (M1/70) from BD Biosciences, eFluor 450 anti-F4/80 (BM8) from eBioscience, rabbit anti-Tmem119 (106-6) from Abcam. For Tmem119, cells were washed and further stained with the secondary antibody AF647 anti-rabbit IgG (catalog: A21245) for 30 min. For intracellular staining of PTEN (138G6, CST) and PE anti-CD68 (eBioscience), the CytoFix/Perm Kit (BD Biosciences) was used according to the manufacture’s protocol. All cytometric data were collected on a LSRII or an Aria III cytometer (BD Biosciences) and were analyzed with FlowJo software (TreeStar). Dead cells were excluded from analysis according to the 7-AAD (Biotium) staining, for intracellular staining zombie yellow (Biolegend) was used to label dead cells.

### RNA-seq analysis of microglia

The library was prepared based on the modified smart-seq2 protocol with modifications to sort 1000 cells instead of single cell. For each group, cerebral cortex isolated from 2-3 male mice were pooled together, microglia were sorted on an Aria III cytometer (BD Biosciences) as 7-AAD^-^CD45^+^CD11b^+^ GFP^+^ cells, 1000 microglia were sorted directly into a 0.2-ml PCR tube containing 6 μl lysis buffer. After that, RNA was reverse transcribed into cDNAs, which were subjected to PCR amplification for 14 cycles. The libraries were sequenced by Illumina HiSeq X Ten System. Sequencing data were mapped to Gencode M20 using Salmon (version 0.8.1) (69) and gene expression matrix was processed using the DESeq2 (version 1.22.2) (70) and visualized by showing the first 3 dimensions calculated by *plotPCA* function. All differential expression analyses were performed by DESeq2 according to the software manual. The standard KEGG pathway genesets are publicly available at https://www.genome.jp/kegg/pathway.html. Gene Set Enrichment Analysis (GSEA) was performed using GSEA_4.2.1 software. The KEGG gene set from MSigDB database was chosen as the reference gene set. Pathways with FDR < 0.05 and p < 0.05 were identified as significant. Bubble plots were generated in R (https://www.r-project.org) using the ‘ggplot’ function in the ggplot2 package. The differentially expressed genes with Log_2_FoldChange (mPTEN^KO^/mPTEN^WT^) and P-Value were used as inputs. The color bar represents Log_2_FoldChange (mPTEN^KO^/mPTEN^WT^) values and the size of each dot represents the P-Value. KEGG pathways of upregulated differentially expressed genes were enriched by using DAVID.

### Immunostaining and morphological analyses of microglia

Brains were removed from mice that were sequentially perfused with PBS and 4% paraformaldehyde, post-fixed overnight, dehydrated in 30% sucrose and embedded in OCT (Sakura). Cryosections (40 μm) were blocked in the staining buffer (PBS containing 5% serum and 0.3% Triton-X) for 2 hrs at room temperature, and incubated with primary antibodies diluted in the same buffer overnight at 4°C. Sections were washed and further stained with the secondary antibody in the dark for 2 hrs at room temperature. The sections were counterstained with DAPI (Biotium), washed and mounted with the ProlongGold Antifade reagent (Invitrogen). Images were collected using an Olympus FV1000 upright microscope (20×) or a Nikon A1Rsi spectral confocal system and Ti-E inverted research microscope (100×). The primary antibodies included rabbit anti-Iba1 (catalog: 019-19741, Wako), goat anti-Iba1 (catalog: ab5076, Abcam), rat anti-CD68 (catalog: MCA1957, Bio-Rad), rabbit anti- VGLUT1 (catalog: 13502, Synaptic Systems), mouse anti-NeuN (catalog: MAB377, millipore), mouse anti-PSD95 (catalog: MAB1596, millipore), rabbit anti Olig2 (catalog: ab109186, Abcam), rabbit anti-S100(catalog: ab52642, Abcam), chicken anti-GFP (catalog: GFP-1010, AVES). Secondary antibodies included Alexa Fluor 488-, 555-, 647-conjugated secondary antibodies against rabbit, goat, mouse, chicken or rat IgG (Life Technologies). For 3D reconstruction, images were captured on a Nikon A1Rsi spectral confocal microscope with 100× lens at a 1024×1024 XY resolution and a 0.2-μm Z-step and analyzed with the Imaris software (Bitplane). Microglia including cellular processes were reconstructed automatically in Imaris, verified manually, and subjected to morphometric analyses by the Filament tracer module. For certain experiments, VGLUT1-positive puncta and the VGLUT1/PSD95 colocalization were quantified using the Spot module of Imaris software. Two spots are considered co-localized when the distance between centers of the two spots is less than the diameter of either spot. A spot diameter of 0.2 μm is empirically determined to be suitable for PSD95 and VGLUT1, and this is applied to automatic analyses of all samples to ensure no human bias was introduced. The inclusion of VGLUT1 in CD68+ lysosomes of microglia were analyzed using the Spot and Surface modules of Imaris software.

### Synaptosome preparation

Mice were perfused with PBS. Isolated cortex pooled from 2 mice per sample were homogenized with a dounce homogenizer in 5ml 0.32M sucrose in 4mM HEPES (pH 7.4) and centrifuged at 1000 g for 10 min at 4°C to remove nuclei and debris. The supernatant was transferred to a new tube and centrifuged at 30000 g for 10 min at 4°C, the crude synaptosome pellet was resuspended in 1 ml of 0.32M sucrose and then layered on top of a discontinuous sucrose gradient (1.0 and 1.2M sucrose) and centrifuged at 120,000 g for 2 hours at 4°C. The synaptosomal fraction was isolated from the gradient interface and diluted into 0.32M sucrose and centrifuged at 200,000 g for 30 min at 4°C. The resulting synaptosomes in the pellet were resuspended in 50 mM HEPES/2mM EDTA solution. The quality of synaptosome preparation was verified by enrichment of synaptic proteins as assessed by Western blotting.

### Western blotting

Proteins from isolated synaptosomes were loaded on 10% SDS-PAGE gel and then transferred to PVDF membranes, blocked in 5% nonfat milk, and incubated with primary antibodies, which included rabbit anti VGLUT1 (Synaptic Systems), mouse anti-PSD95 (Millipore), mouse anti-Gephyrin (Synaptic Systems), rabbit anti GluN2B (Synaptic Systems), rabbit anti-Synaptophysin (Abcam) overnight at 4°C. Lysates from microglia were incubated with the primary antibody rabbit anti PTEN (Cell Signaling Technology). Membranes were then incubated with HRP-conjugated secondary antibodies, and immunoblots were developed with enhanced chemiluminescence (Thermo Fisher Scientific). Images were analyzed with ImageJ software.

### *Ex vivo* phagocytosis assay

Microglia freshly isolated from P7 mice were rested in DMEM (Thermo Fisher Scientific, Gibco) supplemented with 2% FBS for 45 min at 37°C and then incubated in the same media containing 0.2 mg/ml pHrodo Zymosan BioParticles (Life Technologies) for 1.5 h at 37°C. After incubation cells were washed and analyzed by FACS.

### Electrophysiological recording

#### Slice preparation

Coronal slices of the auditory cortex (AC) were obtained from juvenile (P14-16) mPTEN^KO(0)^ and mPTEN^WT(0)^ mice. Mice were anesthetized with sodium pentobarbital (50 mg/kg, i.p.). After mice showed no reflex to foot pitch, juvenile mice were decapitated directly, while young adult mice were perfused with ice-cold sucrose-based ACSF (NaCl was replaced by equiosmolar sucrose, i.e., 213 mM) transcardially before decapitation. The brains were dissected out and immersed in the same solution. Slices with a thickness of 250 μm were cut in this sucrose-based solution with a vibratome (VT 1200S, LEICA). After slicing, they were immediately transferred to an incubation chamber filled with normal ACSF (see below) and maintained at 34.5°C for 30-40 minutes and then at room temperature before use. For recording, individual slices were transferred to a recording chamber perfused with normal ACSF at 34.5°C. An infrared differential interference contrast (IR-DIC) microscope (BX-51WI, Olympus) was used for visualization of individual cells in the slice. The normal ACSF contained (in mM) 126 NaCl, 2.5 KCl, 2 MgSO_4_, 2 CaCl_2_, 26 NaHCO_3_, 1.25 NaH_2_PO_4_ and 25 dextrose (315 mOsm, pH 7.4). To record the mEPSC, we added tetrodotoxin (TTX, 1μM) and picrotoxin (PTX, 50 μM) to the normal ACSF to block Na^+^-action potentials and GABA_A_ receptor mediated inhibitory synaptic transmission, respectively. To evoke EPSCs mediated by non-NMDA receptors, we added PTX (50 μM) as well as AP-5 (50 μM, an NMDA receptor antagonist). All solutions were equilibrated with 95% O_2_ and 5% CO_2_.

#### *In vitro* whole-cell recordings

Layer 5 pyramidal cells (PCs) with apparent apical dendrites were identified through IR-DIC optics. We performed whole-cell recordings from PCs using patch pipettes with an impedance of 4-7 MΩ when filled with Cs^+^ internal solution containing (in mM) CsCH_3_SO_3_ 138, CsCl 3, MgCl_2_ 2, Na_2_ATP 2, HEPES 10, EGTA 0.2 (pH = 7.4, Osm = 289). In some experiments, biocytin (0.2%) was also added to the internal solution for *post hoc* staining. Voltage-clamp recordings were achieved using a MultiClamp 700B amplifier (Molecular Devices). We used Power1401-3A together with Spike2 (version 10, Cambridge Electronic Design) for data acquisition. Current signals were filtered at 10 kHz and sampled at 25 kHz. The mEPSCs were recorded at a holding potential of -70 mV. A segment of 60-second current trace after the initial 120-second recording was included for analysis. The evoked EPSCs were also recorded at -70 mV. Electrical stimulations (100 μs in duration, 5-10 μA in amplitude) were delivered to the neighboring tissue of recorded PCs at layer 5 (100 μm away) using a similar patch pipette filled with ACSF. For each cell, we obtained 15 paired EPSCs at 50 Hz. The paired-pulse stimulation was applied every 10 seconds.

All data was acquired and analyzed by experimenters blind to animal groups. To obtain properties of mEPSCs, we used Minianlaysis (Synaptosoft) to detect and analyze individual mEPSC events semi-automatically. The mEPSC threshold was set at 5 pA. A waveform with rising time and decay time constant less than 2 and 8 ms, respectively, was considered as an mEPSC event. The paired-pulse ratio was the amplitude ratio of paired EPSCs (i.e., EPSC_2_/EPSC_1_). Statistics was performed with Prism (version 6, GraphPad). To compare between groups, we obtained data from at least 8 cells from different animals (at least 3 mice). Data were presented as median together with the interquartile range (IQR) and 99% tails. For the comparison of two independent observations, unpaired Student’s t-test (two tailed) was used for data that passed the normality test; non-normal data were compared using Mann-Whitney test.

### Statistics

The sample size was not statistically pre-determined but empirically estimated to be sufficient in accord with standards in the field. No randomization in our animal experiments was necessary, because only age- and sex-matched mice of different genotypes were compared. Experimenters were blinded to the mouse genotype during behavioral tests and electrophysiological recordings. Statistical analyses were performed using Graphpad Prism. Unpaired Student’s t tests were used to compare different groups unless indicated otherwise. Specifically, paired Student’s t tests were performed to analyze data collected in NORT, three-chamber tests (**Figures 1C-E**, **Figures 4D-F**) and *ex viv*o phagocytosis assay (**Figure 3C**). Mann–Whitney U-tests were performed to analyze data that show skewed distributions, including the amplitude and area of mEPSC in P14 mice (**Figure 5L**) collected by whole-cell recording in brain slices.

## Data availability statement

The datasets presented in this have been deposited at the Genome Sequence Archive under the accession number CRA005932 and are publicly available.

## Ethics statement

The animal study was reviewed and approved by Tsinghua Institution Animal Care and Use Committee.

## Author contributions

HQ and KY conceptualized the study. XZ conducted a majority of the experiments and designed parts of the study. JCW conducted behavioral experiments and together with RZ Western blotting analyses. LL and ZS under the supervision of YS conducted a majority of *in vitro* recording. YL under the supervision of ML conducted part of *in vitro* recording. JY under the supervision of JBW conducted RNA-seq analysis. All authors contributed to data interpretation., HQ supervised the work together with KY, and wrote the paper with XZ and KY. All authors contributed to the article and approved the submitted version.

## Supplementary material

Supplementary Figure 1 Validation of postnatal *Pten* deletion in mPTEN^(0)^ microglia and quantitation of neuron, astrocyte, oligodendrocyte densities. **(A)** Histograms of intracellular PTEN staining of mPTEN^WT(0)^ and mPTEN^KO(0)^ microglia at indicated time points after birth. **(B)** Western blotting of PTEN protein expression in FACS-sorted microglia from mPTEN^WT(0)^ and mPTEN^KO(0)^ mice at indicated time points. In each experiment, P7 microglia of each genotype were pooled from 3-5 mice, microglia of each genotype at 2 to 3 months were pooled from 2 mice. **(C, D)** Co-staining for EYFP (anti-GFP) and different cell-specific markers (Iba1: microglia; S100: astrocyte; Olig2: oligodendrocyte; NeuN: neuron) in S1 **(C)** and Au1 **(D)** cortices of 2- to 3-month-old mPTEN^WT(0)^ mice. **(E)** Numbers of neurons, astrocytes and oligodendrocytes per field (0.4 mm^2^). Each dot represents a mouse (quantitated in n=3 fields per mouse), and lines denote mean±s.e.m. (N=3, 3, 3 mice).

Supplementary Figure 2 Expression of C1q protein in cortex. **(A)** Representative images of C1q staining in the somatosensory cortex of mPTEN^WT(0)^ and mPTEN^KO(0)^ 2-3-month mice. **(B)** Quantification of MFI of C1q signals. Each dot represents a mouse (quantitated in n=4 fields per mouse), and lines denote mean±s.e.m. (N=4, 4 mice). ** <0.01 by t test.

Supplementary Figure 3 Gene set enrichment analysis and KEGG pathway analysis. **(A-C)** Enrichment plots of indicated gene sets in genes differentially expressed between mPTEN^WT(0)^ and mPTEN^KO(0)^ mice at P7 **(A)**, P14 **(B)**, and 2 to 3 months after birth **(C)**. ES, enrichment score; NES, normalized enrichment score; FDR, false discovery rate. **(D)** KEGG pathway enrichment analysis of the upregulated DEGs from P7, P14 and 2 to 3 months mPTEN^KO(0)^ microglia compared to the age matched mPTEN^WT(0)^ control. (FDR<0.05 & Padj<0.05)

Supplementary Figure 4 Transcriptome analysis of mPTEN^(14)^ microglia. **(A)** Transcriptomic PCA analyses of sort-purified cortical microglia from mPTEN^(14)^ mice of 2 to 3 months of age, together with mPTEN^(0)^ mice at P7, P14 and 2-3 months (data in Fig 2). **(B)** A bubble plot showing differential expression (or lack of it) of complement genes. Bubble color, Log_2_FC=Log_2_(mPTEN^KO(0)^/mPTEN^WT(0)^); bubble size, levels of statistical significance defined by *P* values; a dash indicates no significant difference observed. **(C)** A bubble plot showing differential expression (or lack of it) of indicated lysosome-related genes, formatted as in **(B, D)** GSEA, showing enrichment of the oxidative phosphorylation pathway in those genes upregulated in mPTEN^KO(14)^. **(E)** KEGG pathway enrichment analysis of the upregulated DEGs from 2 to 3 months mPTEN^KO(14)^ microglia compared to the age matched mPTEN^WT(14)^ control. (FDR<0.05 & Padj<0.05). (**F**) A bubble plot showing differential expression (or lack of it) of indicated DAM signature genes, formatted as in **(B, G)** A bubble plot showing differential expression (or lack of it) of indicated mitochondria-related genes, formatted as in **(B)**.

Supplementary Figure 5 Neuronal analysis of mPTEN^(14)^ mice. **(A, B)** Western blotting of presynaptic proteins (VGLUT1, synaptophysin), postsynaptic proteins (PSD95, GluN2B), and inhibitory synaptic protein Gephyrin in the 2-to-3-month cortex of mPTEN^WT(14)^ and mPTEN^KO(14)^ mice **(A)** and quantification of their actin-normalized expression levels **(B)**. Bar graphs show mean±s.e.m. of the three (Exp. #1, #2, #3) experiments. **(C, D)** Representative images of VGLUT1 staining of the S1 and Au1 cortex of mPTEN^WT(14)^ and mPTEN^KO(14)^ mice **(C)**, and scatter plots showing densities of VGLUT1 puncta in S1 and Au1 cortex **(D)**. **(E, F)** Representative images of VGLUT1 and PSD95 staining of the S1 and Au1 cortex of mPTEN^WT(14)^ and mPTEN^KO(14)^ mice, with VGLUT1/PSD95 co-localized puncta highlighted with circles **(E)**, and scatter plots showing densities of VGLUT1/PSD95 puncta in the S1 and Au1 mPTEN^(14)^ cortex **(F)**. Data were collected from 3 mice of 2-3 months of age per genotype, 5 fields of S1 and 5 fields of Au1 cortex per mouse. Each symbol is one field, and lines denote mean ± s.e.m.

Supplementary Table 1 The differentially expressed genes between mPTEN^WT(0)^ and mPTEN^KO(0)^ microglia isolated from P7, P14 and 2-3 month mice.

Supplementary Table 2 List of genes belonging to KEGG pathways including oxidative phosphorylation, AD and PD.

Supplementary Table 3 The differentially expressed genes between mPTEN^WT(14)^ and mPTEN^KO(14)^ microglia isolated from 2-3 month mice.
