# Supplementary figures and images for: Microglial Pten safeguards postnatal integrity of the cortex and sociability
Source: Front Immunol. 2022 Dec 14;13:1059364. doi: 10.3389/fimmu.2022.1059364 (PMC9795847; doi:10.3389/fimmu.2022.1059364)

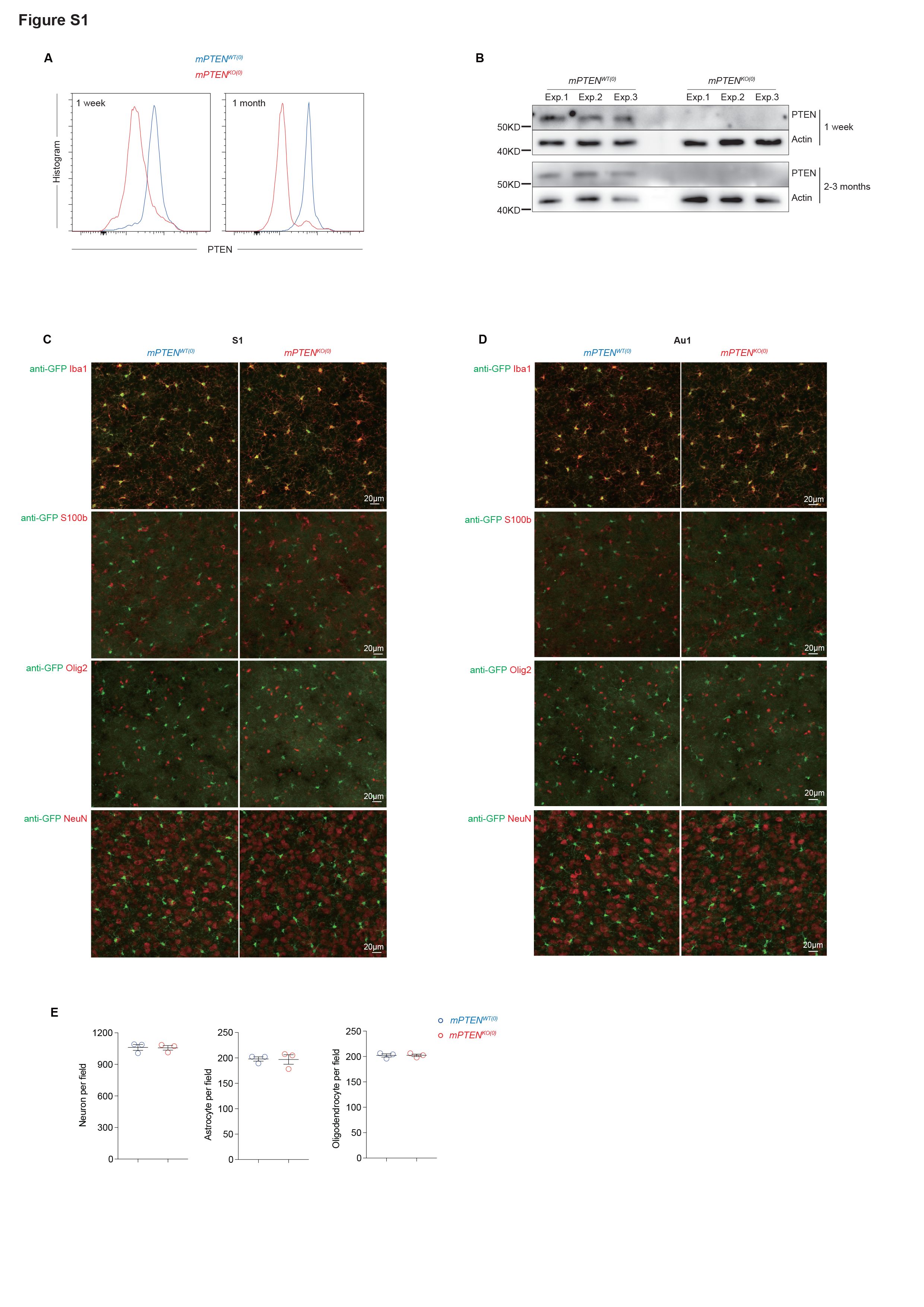

Supplement: Supplementary Figure 1 — Validation of postnatal Pten deletion in mPTEN(0) microglia and quantitation of neuron, astrocyte, oligodendrocyte densities. (A) Histograms of intracellular PTEN staining of mPTENWT(0) and mPTENKO(0) microglia at indicated time points after birth. (B) Western blotting of PTEN protein expression in FACS-sorted microglia from mPTENWT(0) and mPTENKO(0) mice at indicated time points. In each experiment, P7 microglia of each genotype were pooled from 3-5 mice, microglia of each genotype at 2 to 3 months were pooled from 2 mice. (C, D) Co-staining for EYFP (anti-GFP) and different cell-specific markers (Iba1: microglia; S100: astrocyte; Olig2: oligodendrocyte; NeuN: neuron) in S1 (C) and Au1 (D) cortices of 2- to 3-month-old mPTENWT(0) mice. (E) Numbers of neurons, astrocytes and oligodendrocytes per field (0.4 mm2). Each dot represents a mouse (quantitated in n=3 fields per mouse), and lines denote mean±s.e.m. (N=3, 3, 3 mice). [file Image_1.tif]

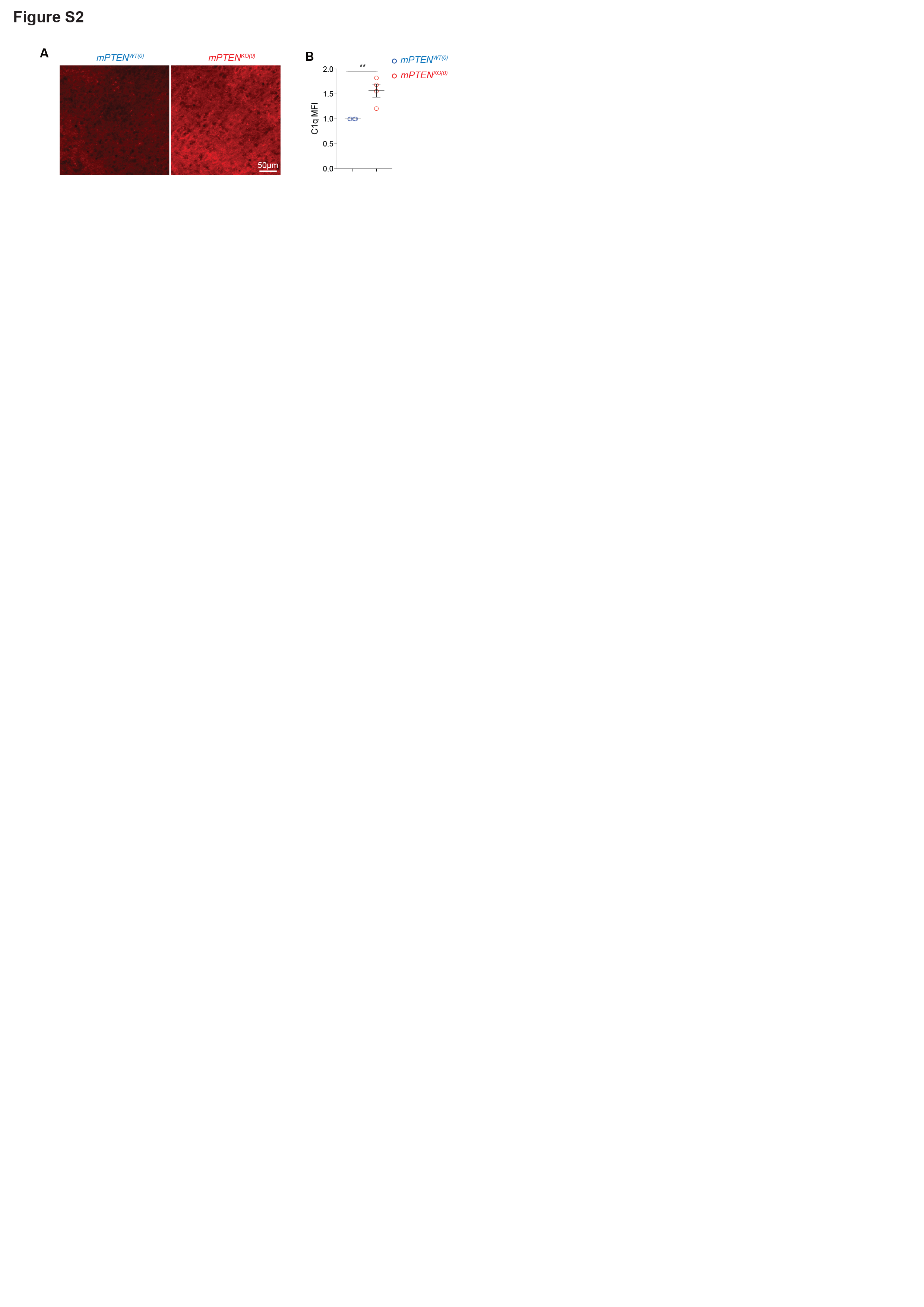

Supplement: Supplementary Figure 2 — Expression of C1q protein in cortex. (A) Representative images of C1q staining in the somatosensory cortex of mPTENWT(0) and mPTENKO(0) 2-3-month mice. (B) Quantification of MFI of C1q signals. Each dot represents a mouse (quantitated in n=4 fields per mouse), and lines denote mean±s.e.m. (N=4, 4 mice). ** <0.01 by t test. [file Image_2.tif]

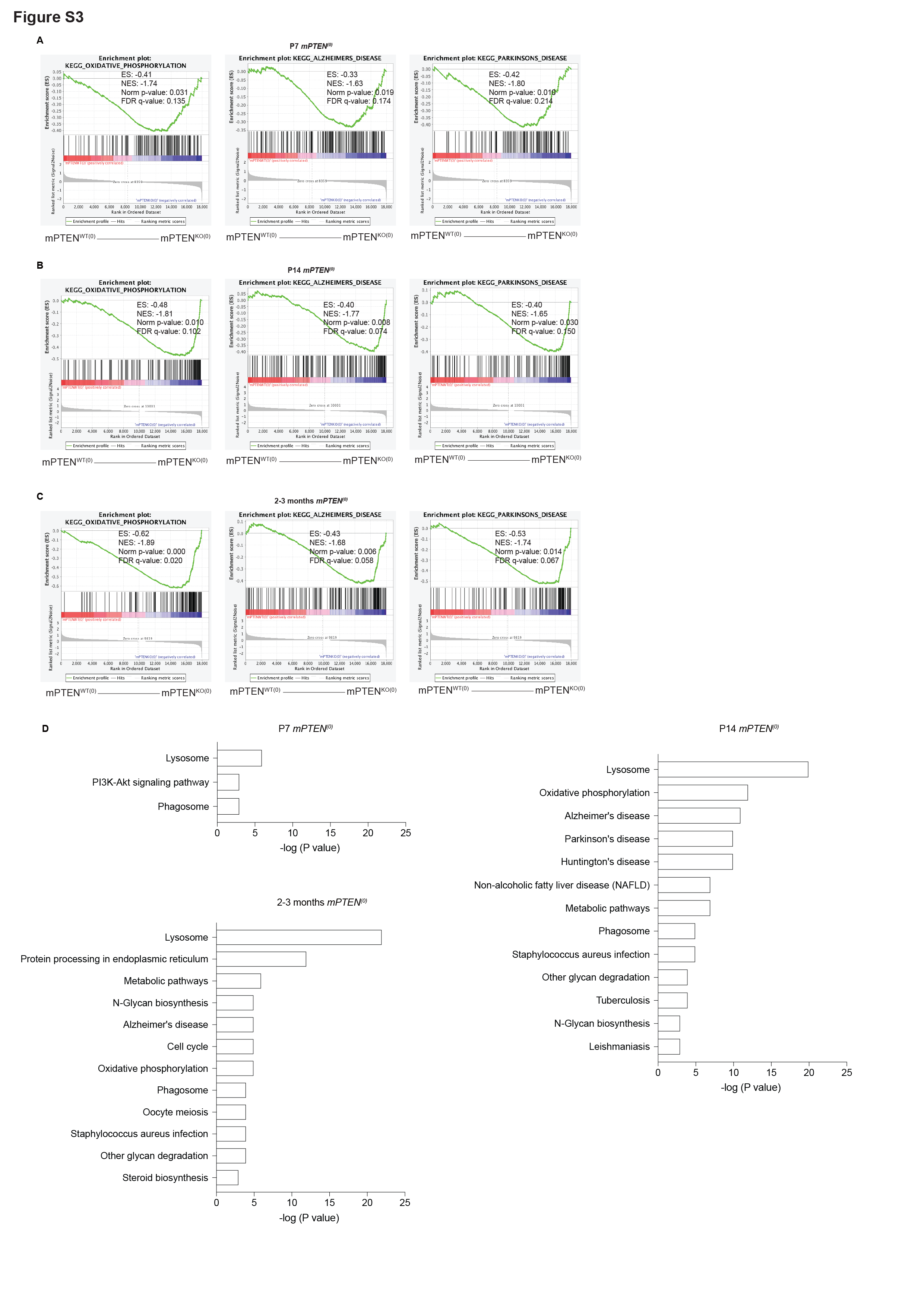

Supplement: Supplementary Figure 3 — Gene set enrichment analysis and KEGG pathway analysis. (A-C) Enrichment plots of indicated gene sets in genes differentially expressed between mPTENWT(0) and mPTENKO(0) mice at P7 (A), P14 (B), and 2 to 3 months after birth (C). ES, enrichment score; NES, normalized enrichment score; FDR, false discovery rate. (D) KEGG pathway enrichment analysis of the upregulated DEGs from P7, P14 and 2 to 3 months mPTENKO(0) microglia compared to the age matched mPTENWT(0) control. (FDR<0.05 & Padj<0.05) [file Image_3.tif]

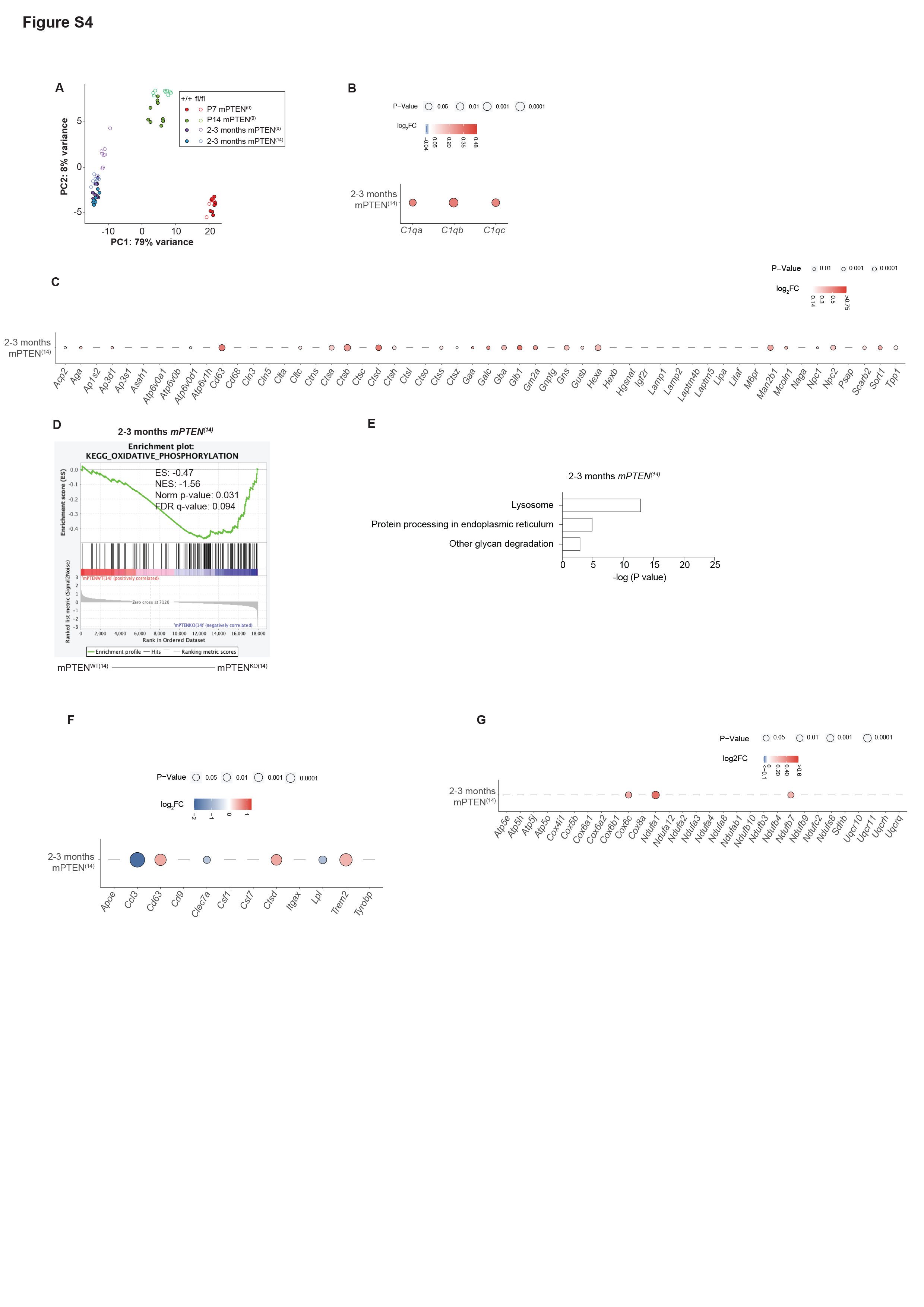

Supplement: Supplementary Figure 4 — Transcriptome analysis of mPTEN(14) microglia. (A) Transcriptomic PCA analyses of sort-purified cortical microglia from mPTEN(14) mice of 2 to 3 months of age, together with mPTEN(0) mice at P7, P14 and 2-3 months (data in Fig 2). (B) A bubble plot showing differential expression (or lack of it) of complement genes. Bubble color, Log2FC=Log2(mPTENKO(0)/mPTENWT(0)); bubble size, levels of statistical significance defined by P values; a dash indicates no significant difference observed. (C) A bubble plot showing differential expression (or lack of it) of indicated lysosome-related genes, formatted as in (B, D) GSEA, showing enrichment of the oxidative phosphorylation pathway in those genes upregulated in mPTENKO(14). (E) KEGG pathway enrichment analysis of the upregulated DEGs from 2 to 3 months mPTENKO(14) microglia compared to the age matched mPTENWT(14) control. (FDR<0.05 & Padj<0.05). (F) A bubble plot showing differential expression (or lack of it) of indicated DAM signature genes, formatted as in (B, G) A bubble plot showing differential expression (or lack of it) of indicated mitochondria-related genes, formatted as in (B). [file Image_4.tif]

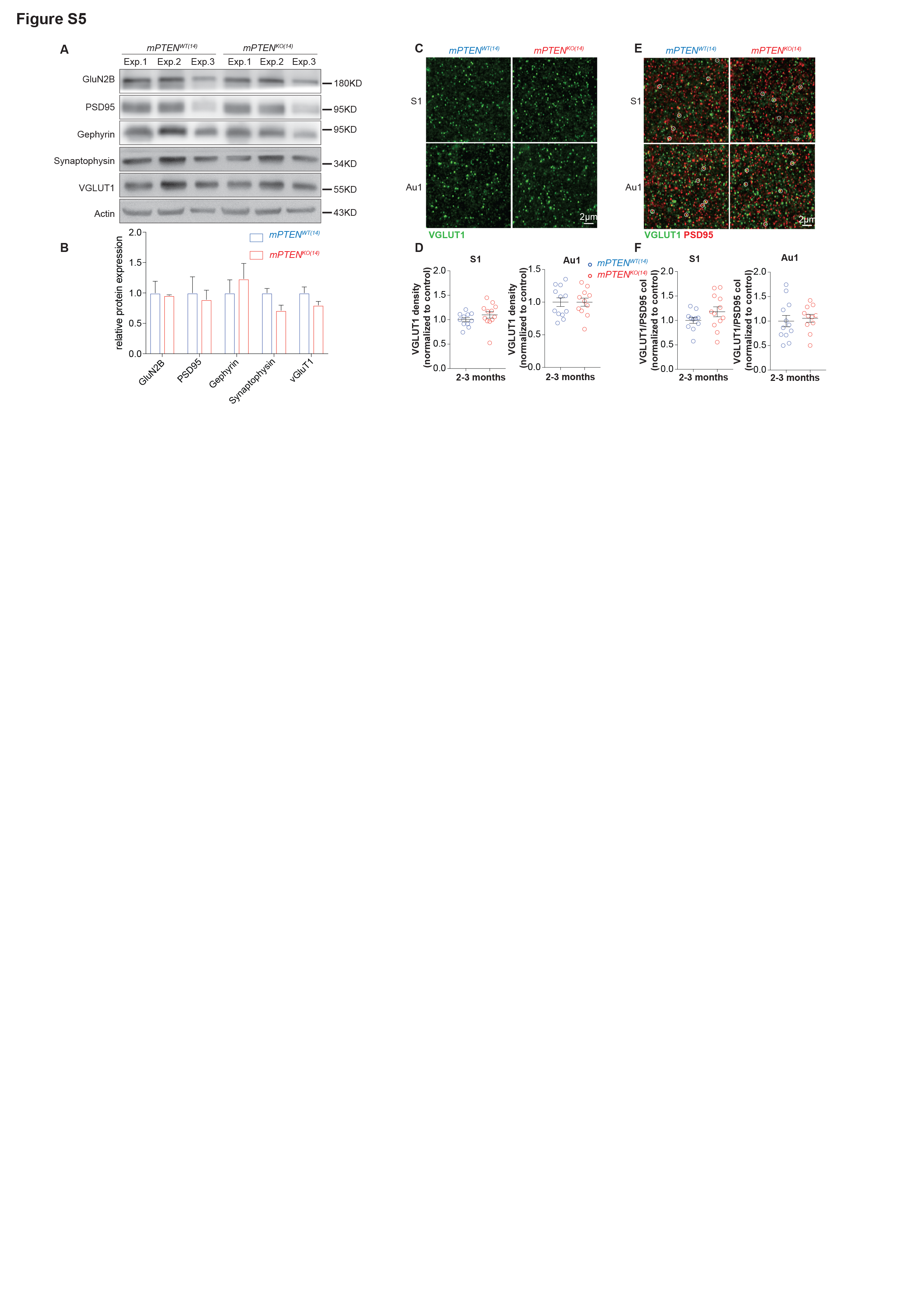

Supplement: Supplementary Figure 5 — Neuronal analysis of mPTEN(14) mice. (A, B) Western blotting of presynaptic proteins (VGLUT1, synaptophysin), postsynaptic proteins (PSD95, GluN2B), and inhibitory synaptic protein Gephyrin in the 2-to-3-month cortex of mPTENWT(14) and mPTENKO(14) mice (A) and quantification of their actin-normalized expression levels (B). Bar graphs show mean±s.e.m. of the three (Exp. #1, #2, #3) experiments. (C, D) Representative images of VGLUT1 staining of the S1 and Au1 cortex of mPTENWT(14) and mPTENKO(14) mice (C), and scatter plots showing densities of VGLUT1 puncta in S1 and Au1 cortex (D). (E, F) Representative images of VGLUT1 and PSD95 staining of the S1 and Au1 cortex of mPTENWT(14) and mPTENKO(14) mice, with VGLUT1/PSD95 co-localized puncta highlighted with circles (E), and scatter plots showing densities of VGLUT1/PSD95 puncta in the S1 and Au1 mPTEN(14) cortex (F). Data were collected from 3 mice of 2-3 months of age per genotype, 5 fields of S1 and 5 fields of Au1 cortex per mouse. Each symbol is one field, and lines denote mean ± s.e.m. [file Image_5.tif]
